# Quality of reporting of outcomes in phase III studies of pulmonary tuberculosis: a systematic review

**DOI:** 10.1186/s13063-018-2522-x

**Published:** 2018-02-21

**Authors:** Laura Jayne Bonnett, Gie Ken-Dror, Geraint Rhys Davies

**Affiliations:** 1 Department of Biostatistics, University of Liverpool, Waterhouse Building, Block F, 1-5 Brownlow Street, Liverpool, L69 3GL UK; 2 Department of Biostatistics and Department of Clinical Infection, Microbiology, and Immunology, University of Liverpool, Waterhouse Building, Block F, 1-5 Brownlow Street, Liverpool, L69 3GL UK; 3 Department of Clinical Infection, Microbiology, and Immunology, University of Liverpool, Ronald Ross Building, 8 West Derby Street, Liverpool, L69 7BE UK

**Keywords:** Tuberculosis, Systematic review, Outcomes, Phase III clinical trials

## Abstract

**Background:**

Despite more than 60 years of clinical trials, tuberculosis (TB) still causes a high global burden of mortality and morbidity. Treatment currently requires multiple drugs in combination, taken over a prolonged period. New drugs are needed to shorten treatment duration, prevent resistance and reduce adverse events. However, to improve on current methodology in drug development, a more complete understanding of the existing clinical evidence base is required.

**Methods:**

A systematic review was undertaken to summarise outcomes reported in phase III trials of patients with newly diagnosed pulmonary TB. A systematic search of databases (PubMed, MEDLINE, EMBASE, CENTRAL and LILACs) was conducted on 30 November 2017 to retrieve relevant peer-reviewed articles. Reference lists of included studies were also searched. This systematic review considered all reported outcomes.

**Results:**

Of 248 included studies, 229 considered “on-treatment” outcomes whilst 148 reported “off-treatment” outcomes. There was wide variation and ambiguity in the definition of reported outcomes, including their relationship to treatment and in the time points evaluated. Additional challenges were observed regarding the analysis approach taken (per protocol versus intention to treat) and the varying durations of “intensive” and “continuation” phases of treatment. Bacteriological outcomes were most frequently reported but radiological and clinical data were often included as an implicit or explicit component of the overall definition of outcome.

**Conclusions:**

Terminology used to define long-term outcomes in phase III trials is inconsistent, reflecting evolving differences in protocols and practices. For successful future cumulative meta-analysis, the findings of this review suggest that greater availability of individual patient data and the development of a core outcome set would be desirable. In the meantime, we propose a simple and logical approach which should facilitate combination of key evidence and inform improvements in the methodology of TB drug development and clinical trials.

**Electronic supplementary material:**

The online version of this article (10.1186/s13063-018-2522-x) contains supplementary material, which is available to authorized users.

## Background

Tuberculosis (TB) remains a major killer amongst infectious diseases with 10.4 million new cases and 1.8 million deaths worldwide in 2015 [1]. Though TB incidence has fallen by an average of 1.5% per year since 2000, this needs to accelerate to a 4–5% annual decline to reach the 2020 milestones of the World Health Organization’s End TB Strategy, which include reducing TB deaths by 90% and cutting new cases by 80% between 2015 and 2030 [2]. Ending the TB epidemic by 2030 is also among the health targets of the newly adopted Sustainable Development Goals [3].

Current treatment for drug-sensitive TB involves administration of four drugs for 6 months, while for multidrug-resistant disease, up to seven drugs for 9 to 24 months may be required. New drugs and regimens are required to shorten treatment duration, reduce toxicity and combat drug resistance but the optimal critical path for novel regimens is not well-defined. Currently, clinical development of a single novel TB drug is expected to take not less than 6 years, while a completely novel combination regimen developed sequentially would require around 20 years or more (http://www.cptrinitiative.org/). Phase III trials aiming to shorten the duration of first-line TB therapy must be large and follow-up prolonged because of the strength of the current comparator in assuring stable cure [4]. Recent such trials have been costly and global capacity for supporting them is limited [5].

Possible outcomes for phase III TB trials have a number of important dimensions. Studies may focus on bacteriological, clinical and/or radiological outcomes, or some combination of the three and these outcomes are typically followed on multiple occasions during and after treatment. The structure of treatment regimens has varied historically with de-intensification after an initial “intensive” phase, a common feature, and the necessary duration of follow-up post-treatment has not been defined. Investigators commonly report apparently simple outcomes such as cure, relapse and death, which may have very different meanings in different studies. This diversity is problematic as, without comparability of outcomes across trials, it is challenging to synthesize evidence effectively and draw the methodological conclusions necessary to inform decision-making for public health and improve the conduct of future trials. This issue has become pressing in light of recent prominent disappointments in phase III studies and with the emerging need in the field to evaluate novel combinations of drugs efficiently [6]. With this in mind, we systematically reviewed the literature of phase III studies in pulmonary TB to determine how outcomes are currently defined, and how commonly they are used in published studies to date.

## Methods

The available literature was searched for randomised controlled trials, or quasi-randomised trials meeting the following inclusion criteria: trials including patients with smear- and culture-positive pulmonary tuberculosis, trials including patients receiving treatment for the first time or with known isoniazid mono-resistant organisms on susceptibility testing, and including regimens containing any combination of the following drugs. Historic first-line treatment regimens with existing clinical data were included. Additionally, any drugs with an active phase II clinical development programme were also included. In particular trials including regimens containing any combination of ethambutol (E), gatifloxacin (G), isoniazid (H), bedaquiline (J), levofloxacin (L), moxifloxacin (M), ofloxacin (O), para-aminosalicylic acid (P), PA-824 (Pa), rifampicin (R), rifabutin (Rb), rifapentine (Rp), streptomycin (S), thiacetazone (T), and pyrazinamide (Z) were considered. There were no specific exclusion criteria in order to keep the review as broad as possible.

PubMed, MEDLINE, EMBASE, CENTRAL and LILACs were searched on 30 November 2017 using a defined inclusive search strategy as follows, without language restrictions. No date restrictions were applied

1. Tuberculosis and clinical trials
2. Rifampicin or isoniazid or pyrazinamide or ethambutol or thiacetazone or para-aminosalicylic acid or streptomycin or rifabutin or rifapentine or levofloxacin or ofloxacin or gatifloxacin or moxifloxacin or bedaquiline or PA-824
3. 1 and 2

Hand searching of reference lists of included studies and relevant reviews was also undertaken to ensure all relevant studies were included. Two authors (LB and GK-D) assessed all studies evaluating either monotherapy or combination regimens for inclusion. Phase III studies were identified on a case-by-case basis according to whether the study reported the primary end of treatment or post-treatment outcomes at an appropriate sample size. Ambiguous cases and any disagreements were resolved by discussion with the third author (GD). Two investigators (LB and GK-D) extracted the data using a piloted data extraction form. Data were summarised using appropriate graphical and numerical summaries in R (version 3.3.2). In the case of multiple publications relating to a single study, the publication with the largest number of reported outcomes was included as the main publication. The associated manuscripts were also checked for additional outcomes, which were additionally reported as though they were part of the main publication. The protocol for this systematic review is available on request. The review was not registered with PROSPERO.

As a sensitivity analysis, we considered Venn diagrams for the reported outcomes in trials published before 1995 and from 1995 onwards. This cut-off was chosen as representing the start of the modern era in TB clinical trials.

## Results

### Characteristics of included studies

**Fig. 1:**
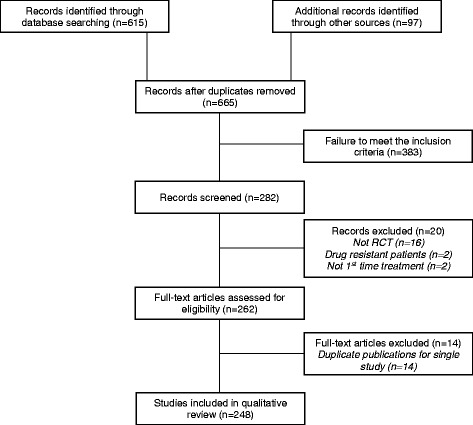
Flow of studies in the review. *RCT* randomised controlled trial

**Fig. 2:**
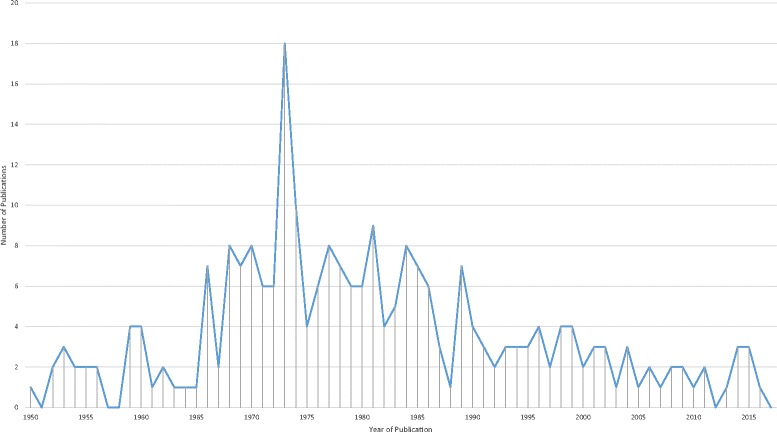
Year of publication of included studies

Figure 1 illustrates the flow of studies through the review. The main reasons for exclusion were failure to meet the inclusion criteria, and study design other than randomised controlled trial. In total, 248 relevant studies were identified and included. The included studies ranged in year of publication from 1950 until 2016 (Fig. 2). Only 45 (18%) of the included studies were published after 1995. Hence the majority of included studies may not be expected to conform to the CONSORT guidelines on reporting of items such as trial design, intervention, participants, and outcomes [7]. Thirty-nine studies were reported in languages other than English. The most frequent alternative languages were Japanese (12 studies), German (eight studies) and French (seven studies) with alternatives including Chinese, Italian, Danish, Russian, Dutch, Portuguese, Romanian and Polish.

**Fig. 3:**
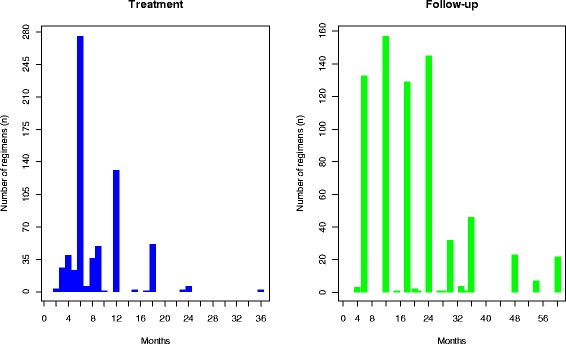
Bar charts illustrating the number of included studies reporting each duration of treatment (*left: blue bars*) and also the duration of follow-up (*right: green bars*)

Figure 3 summarises the duration of treatment across studies and the extent of follow-up. The most frequent treatment durations were 6 and 12 months. Some studies only treated patients for 2 months [8–10] while others tested treatment plans lasting 36 months [11, 12]. Follow-up after treatment was completed was most frequently undertaken at 6, 12, 18, and 24 months. The shortest duration of follow-up was 4 months [13] with some studies opting for 60 months of follow-up (e.g. [14–16]). During pilot data extraction, it was noted that definitions of treatment failure and relapse used in the reports were often inconsistently related to stage of treatment. For this reason, for the purposes of the review, outcomes were classified as “on-treatment” if treatment was continuing at the time of their evaluation, “off-treatment” if treatment had already been completed and “end of treatment” if they represented the final on-treatment evaluation.

### Definitions of outcomes

There was wide variation in the definition and type of outcomes reported in the included studies. Nearly all reported multiple outcomes without any clear distinction between primary and secondary outcomes. Therefore all the outcomes included in each study were considered (*n* = 1378), and are summarised in Additional file 1. The most frequently reported outcomes were the proportion of participants experiencing a “relapse” (positive bacteriological tests in the “off-treatment” phase) (136 out of 1378 reported outcomes, 10%), improvement or change in radiograph (*n* = 100, 7%), and death from all or unspecified causes (*n* = 90, 7%). Adverse events were also frequently reported (*n* = 179, 13%).

The reported outcomes can be broadly categorised into bacteriological (for example negative or positive mycobacterial cultures on various media), radiological (for example number of cavities seen on chest radiograph) and clinical (for example death), evaluated on or off treatment. A Venn diagram summarising the number of trial reports including each of these categories of outcomes can be seen in Additional file 2; 27 studies reported only off-treatment outcomes while 106 studies reported only on-treatment outcomes. The sensitivity analysis considering the reported outcomes in trials published before 1995 and from 1995 onwards can be seen in Additional file 3. It appears that in recent years trials have been less reliant on radiological results.

### Bacteriological outcomes

A total of 218 (82%) studies reported a bacteriological endpoint on treatment whilst 139 (52%) reported such an endpoint off treatment. There was a lack of consistency in whether sputum smear results were characterised as positive or negative and considerable variation in the time points at which relapse was assessed. Culture or smear status were more frequently reported as negative (102 of 1378 reported outcomes, 7%) than positive (24, 2%).

Some studies considered “relapse” on treatment [17], while others considered it at various time points off treatment such as 12 [18], 18 [19], 24 [20], 36 [21], and 60 months [16]. The definition of relapse also varied across studies, some considering relapse as defined by smear results [22], by both culture and smear results [22], or according to radiographic confirmation alone [23]. In some modern trials, relapse was adjusted for molecular typing methods [24]. Similar variability was observed among definitions of treatment failure [22]. Among bacteriological outcomes, the number of cultures obtained also varied from one culture at one time point (e.g. [25–27]) to multiple cultures at one time point, sometimes within one study (e.g. [28, 29]). Some studies adopted an intention-to-treat approach to analysis and included patients with missing or contaminated culture results, while others used a per protocol approach and excluded these patients. Poor-quality reporting and high risk of bias meant it was mostly impossible to distinguish these situations.

### Death

A total of 126 (51%) studies reported an outcome related to death (either on treatment, or off treatment). Death was classified in multiple ways ranging from all causes [30], through non-TB [31], to TB [32], assessed at a number of time points, and expressed as a variety of endpoints ranging from proportion to time to event. Fifty-three (21%) studies attempted to attribute cause of death to TB.

### Composite endpoints

A subset of the included studies considered an explicitly composite endpoint of “unfavourable” as against “favourable” [33]. “Unfavourable” in modern trials characteristically included bacteriologically or clinically defined treatment failure or relapse [34], death from all causes (with the possible exception of trauma) while on treatment, death from TB in the off-treatment phase, failure to complete the treatment regimen within a defined timeframe and/or withdrawal from the study before the end of treatment [13]. However, there were often potentially significant differences in the exact definition and implementation of the “unfavourable” outcome, even among the most recently conducted trials evaluating very similar treatment regimens.

## Conclusions

Our systematic review identified considerable and potentially important variation among reported outcomes and their definitions among phase III trials of treatment for TB. These differences were apparent across a number of key dimensions and details. While the review necessarily reflects the evolution of clinical trials in TB over more than six decades, our findings have implications for the design and conduct of such studies in the future.

It is possible that the definition of outcomes could be related to progress and trends within clinical guidelines in the pulmonary TB area (e.g. [35]). Such guidelines may have contributed to some changes in reported outcomes over time. However, the guidelines are focused on programmatic treatment strategies and diagnostic methods rather than outcomes to be reported within clinical trials.

Overall, studies were frequently hampered by the use of ambiguous or incompletely defined terms such as “treatment failure” and “relapse”, which often appeared to embody unstated clinical or temporal assumptions. Over time, objective and usually blinded bacteriological assessments have been increasingly prioritised by TB trialists, particularly during the on-treatment phase while off-treatment outcomes, although similarly based on bacteriology, often also have a clinical and/or a radiological dimension which is not always explicit. In addition, the historical concept of “relapse” has been complicated by the recognition of reinfection enabled by novel molecular techniques. There was inconsistency in selection of the time points at which the outcomes were considered, the precise requirements for declaring bacteriological assessments negative or positive and in the total duration of off-treatment follow-up. While on-treatment outcomes would logically be reported as conversion to negative status and off-treatment outcomes as conversion to positive status, this distinction was not consistently applied across trials. In addition few trials clearly defined or reported per-protocol or intention-to-treat analysis populations or losses to follow-up.

To avoid some of these problems, trialists have increasingly wished to explicitly combine several endpoints into a composite endpoint which better reflects the total performance of a treatment regimen over the entire course of treatment and follow-up, and avoids the conditional linkage between on-treatment and off-treatment outcomes (absence of a poor on-treatment outcome being a precondition for off-treatment follow-up). Historically, these composite endpoints have been described as “favourable”/“unfavourable”. While this is a logical approach, in practice the scope of this composite endpoint may vary significantly, even between recent trials and the importance of some components can vary between trial settings, particularly mortality in study populations with a high rate of HIV seropositivity. Without closer harmonisation of such composite endpoints, it will remain important to continue to clearly report the disaggregated component outcomes as well as the composite in order to facilitate appropriate meta-analysis.

In a prior systematic review of methodology in phase II studies in TB, we identified multiple competing approaches and analysis methods, which may be equally valid in an exploratory context [36]. Phase III outcomes, however, are confirmatory in nature and require broader consensus from both trialists and regulators. This review suggests that simple measures could improve the quality of reporting of phase III studies and also highlights the need to develop a core outcome set for future pivotal trials in TB. This would provide a set of clearly defined outcomes to be reported in each study [37], but which would not be exhaustive giving trialists the freedom to report other outcomes of specific interest. The Core Outcome Measures in Effectiveness Trials (COMET) Initiative brings together people interested in the development and application of agreed standardised sets of outcomes. Thus our work is relevant in a broader setting than just trials within tuberculosis. Agreement on a meaningful core outcome set would make it easier for future trials to be compared, contrasted and combined in systematic reviews, ultimately facilitating understanding and accelerating improvement of treatment strategies for patients with TB. This work is currently ongoing for phase II studies within TB following a recent systematic review of outcomes [36]. However, pending development of, and agreement on, such a core outcome set, we suggest some interim recommendations for reporting of future phase III trials:

Endpoints should be explicitly referred to in terms of their timing in relation to the end of treatment. Rather than the traditional and sometimes ambiguous terms “treatment failure” and “relapse”, bacteriological, clinical and/or radiological outcomes would be more clearly described as “on-treatment” or “off-treatment”.

Composite primary endpoints, for example, “unfavourable” or “favourable” should be clearly defined in trial reports and appropriately disaggregated into their components in relevant analyses.

Reporting of bacteriological outcomes should clearly specify the definition of bacteriological conversion (whether negative or positive, including whether more than one time point is required and whether samples are to be replicated at each time point) and report the reasons for missing cultures (not obtained, contaminated). While for phase II it seems most logical to report outcomes as conversion to culture negativity, for the purposes of reporting combined endpoints in phase III trials, it is most consistent to report the positive on- and off-treatment outcomes together to avoid conditionality.

Finally, where these recommendations cannot be met, availability of individual patient data could facilitate appropriate re-analysis and combination of similar outcomes wherever possible thus enabling and reducing heterogeneity in future meta-analyses. Improving the quality and interpretation of this evidence base will be critical in providing the best possible information on the equipoise and design of future clinical trials in TB.

## Additional files

Additional file 1: Reported outcomes. (DOCX 17 kb)

Additional file 2: Venn diagram summarising all reported outcomes. (PPTX 71 kb)

Additional file 3: Venn diagrams summarising all reported outcomes according to the sensitivity analysis (i.e. pre- and post-CONSORT). (PPTX 80 kb)

## Abbreviations

E: Ethambutol
G: Gatifloxacin
H: Isoniazid
J: Bedaquiline
L: Levofloxacin
M: Moxifloxacin
O: Ofloxacin
P: Para-aminosalicylic acid
Pa: PA-824
R: Rifampicin
Rb: Rifabutin
Rp: Rifapentine
S: Streptomycin
T: Thiacetazone
TB: Tuberculosis
Z: Pyrazinamide
