# Supplementary material for: Quality of reporting of outcomes in phase III studies of pulmonary tuberculosis: a systematic review
Source: Trials. 2018 Feb 21;19:134. doi: 10.1186/s13063-018-2522-x (PMC5822642; doi:10.1186/s13063-018-2522-x)
Supplement: Supplementary file 1 — Reported outcomes. (DOCX 17 kb) [file 13063_2018_2522_MOESM1_ESM.docx]

Additional Table 1: Reported outcomes

| **Outcome** | **Definition** | **No. studies reporting outcome on treatment** | **No. studies reporting outcome off treatment** |
| --- | --- | --- | --- |
| Treatment Outcomes  Treatment completion  Treatment changes  Treatment change reason  Treatment outcome  Treatment duration  Time to treatment change  Default rate  Cost of treatment  Alternative therapies | Number completing (or not completing) treatment including those defaulting  Number changing treatment including adding treatment  Reason for treatment change  Including collapse and crush therapy | 11  9  1  11  7  1  2  1  2 | 14  2  1  14  6  0  1  1  2 |
| Bacteriological  Number culture negative  Number culture positive  Culture status/result  Culture conversion  Number smear negative  Number smear positive  Sputum conversion  Sterilisation rate  Bacteriological change  Acid Fast Bacilli count  Bacterial Quiescence  Mean bacilli index  Sputum character/volume  Negative microscopy  Predictive value of positive smear  Smear status  Phage typing  RFLP typing | Time to and rate of culture conversion  Time to, proportion, and rate of sputum conversion  Change/conversion (proportion & rate)  Or Quiescent disease  Change in  Change in | 83  17  14  29  17  7  40  1  12  10  8  2  2  1  0  3  0  0 | 42  6  9  19  4  1  21  0  12  1  6  0  0  1  1  0  1  1 |
| Death  Death (all causes)  Death (not TB)  Death (TB)  Time to death  Time to TB death  Mortality rate  Survival probability | Or not specified  All causes or not specified | 83  18  44  10  1  0  1 | 54  20  28  4  1  1  2 |
| Failure  Number failing  Time to failure  Number failing/relapse  Time to failure/relapse  Reason for unfavourable outcome | Treatment or bacteriological failure  Time to or rate of failure  Composite outcome (inseparable)  Or unfavourable outcome in general | 35  3  3  7  1 | 38  2  5  6  1 |
| Relapse  Proportion relapsing  Time to relapse  Relapse probability  Retreatment | Including x-ray proven relapse  Time to, or rate of, relapse/recurrence  Number requiring retreatment for relapse or recurrence | 115  19  0  1 | 128  25  1  1 |
| Response including Cure  Response  Status/Classification  Cure  Cure rate  Favourable outcome  Time to favourable outcome | Bacteriological or treatment  Disease and/or bacteriological and/or patient  Proportion | 16  71  2  1  3  0 | 12  46  1  1  4  1 |
| X-rays  Radiograph  Cavitation  Cavity closure rate  X-ray resolution rate  Number of cavities  Involved lung zones  Cavity condition  Radiographic characteristics  Compaction | Improvement/Change in  Improvement/Change in  Rate or proportion  Rate or proportion  Cavities, lesions or shadows  Condition or index | 96  35  3  1  4  1  1  1  1 | 39  12  0  0  1  0  1  0  0 |
| Patient related  Clinical improvement  Weight gain/change  Temperature change  Severity  Patient satisfaction  Respiratory function  Hospitalisation  Temporary disability | Improvement or change in clinical assessment  Improvement/change in  Japanese severity scoring system  Duration  Duration | 23  23  6  2  1  1  1  0 | 11  6  0  1  0  0  1  1 |
| Resistance  Drug resistance  R-dependent antibodies | Resistance/sensitivity/susceptibility | 77  1 | 37  0 |
| Test results  Liver function  Haemoglobin  ESR value  Laboratory tests  Blood sedimentation rate  Blood glucose  Prolongation of QT  Urine test | Function or dysfunction including SGOT and SGPT  Concentration or result  Erythrocyte sedimentation rate value  Including blood tests (but without specific haemoglobin value) | 17  3  8  3  2  1  1  1 | 6  1  4  0  1  1  1  0 |
| Adverse events  Adverse events  Time to adverse events  Management | Proportion of patients with adverse events (including serious)  Management of adverse events | 167  2  1 | 98  0  0 |
| Other  Prevalence  Extent of disease  Diabetes score | Prevalence of, or change in prevalence of TB and/or tuberculin predictors | 3  1  1 | 3  0  1 |

NB: Some studies reported both on and off treatment outcomes.
