# Supplementary material for: Quality of reporting of outcomes in phase III studies of pulmonary tuberculosis: a systematic review
Source: Trials. 2018 Feb 21;19:134. doi: 10.1186/s13063-018-2522-x (PMC5822642; doi:10.1186/s13063-018-2522-x)
Supplement: Supplementary file 2 — Venn diagram summarising all reported outcomes. (PPTX 71 kb) [file 13063_2018_2522_MOESM2_ESM.pptx]

## Slide 1
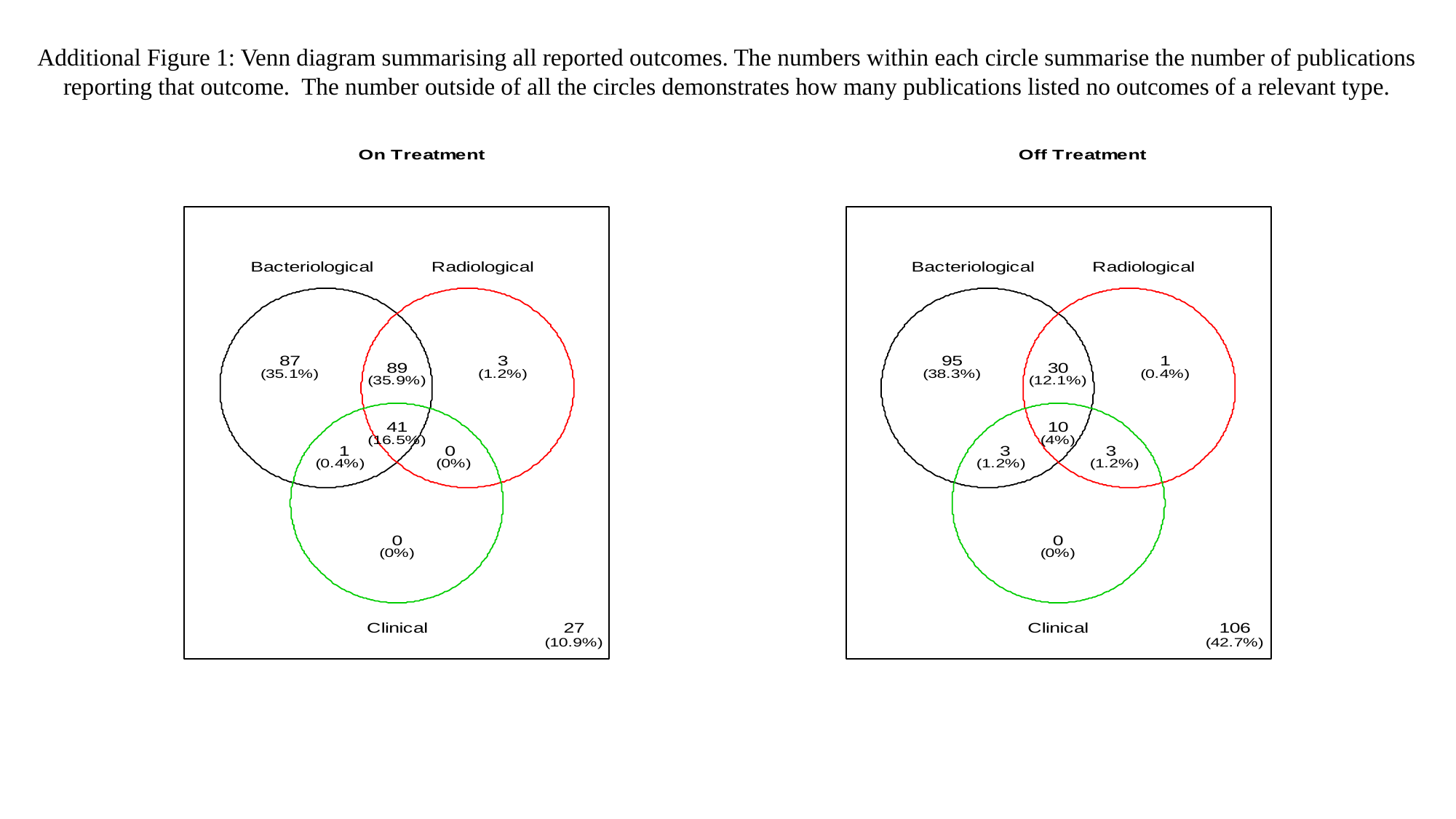

Additional Figure 1: Venn diagram summarising all reported outcomes. The numbers within each circle summarise the number of publications reporting that outcome. The number outside of all the circles demonstrates how many publications listed no outcomes of a relevant type.
