# Supplementary material for: Quality of reporting of outcomes in phase III studies of pulmonary tuberculosis: a systematic review
Source: Trials. 2018 Feb 21;19:134. doi: 10.1186/s13063-018-2522-x (PMC5822642; doi:10.1186/s13063-018-2522-x)
Supplement: Supplementary file 3 — Venn diagrams summarising all reported outcomes according to the sensitivity analysis (i.e. pre- and post-CONSORT). (PPTX 80 kb) [file 13063_2018_2522_MOESM3_ESM.pptx]

## Slide 1
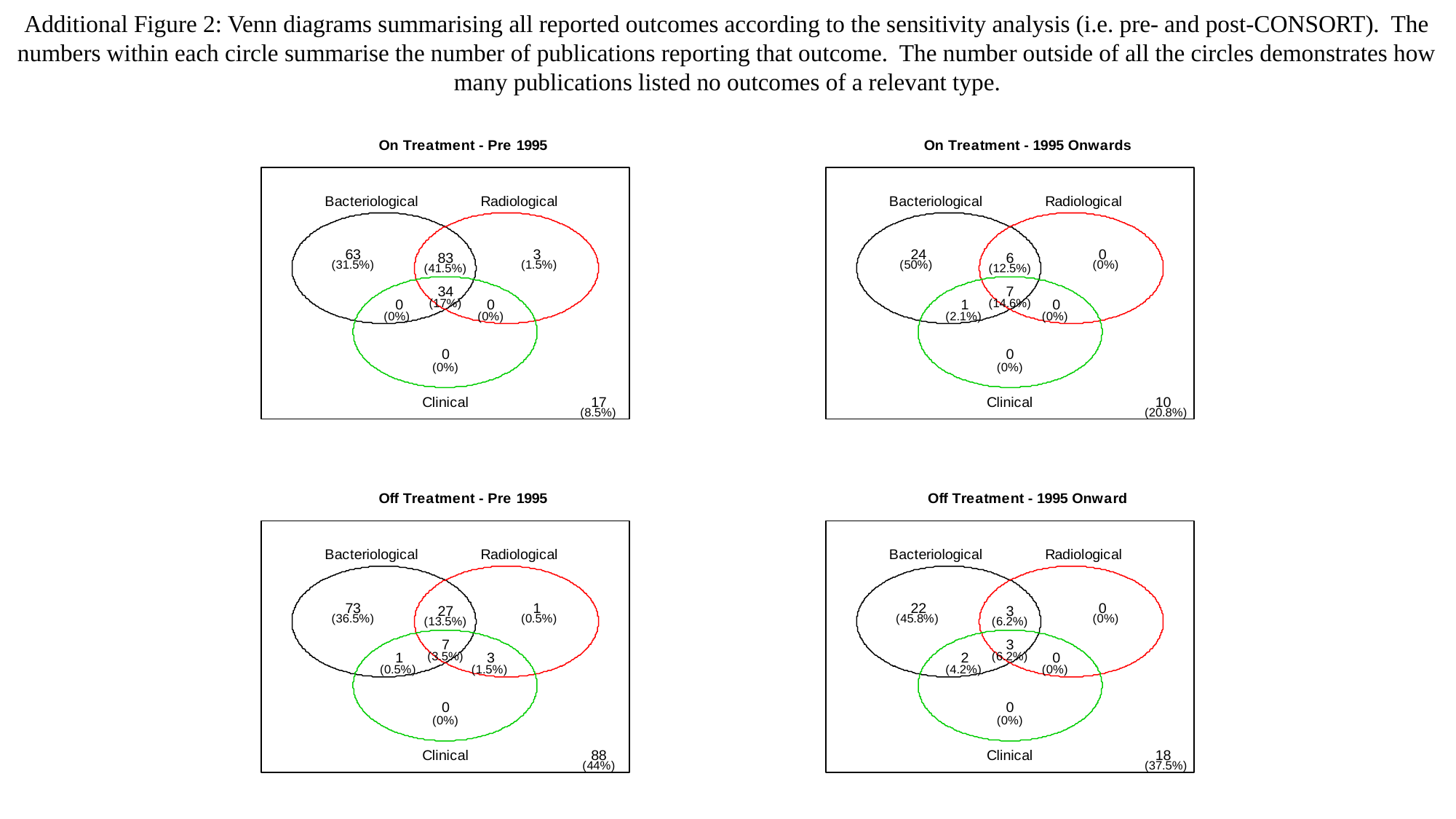

Additional Figure 2: Venn diagrams summarising all reported outcomes according to the sensitivity analysis (i.e. pre- and post-CONSORT). The numbers within each circle summarise the number of publications reporting that outcome. The number outside of all the circles demonstrates how many publications listed no outcomes of a relevant type.
